# AQP4ex is crucial for the anchoring of AQP4 at the astrocyte end-feet and for neuromyelitis optica antibody binding

**DOI:** 10.1186/s40478-019-0707-5

**Published:** 2019-04-01

**Authors:** Claudia Palazzo, Cinzia Buccoliero, Maria Grazia Mola, Pasqua Abbrescia, Grazia Paola Nicchia, Maria Trojano, Antonio Frigeri

**Affiliations:** 1 Department of Basic Medical Sciences, Neurosciences and Sense Organs, School of Medicine, University of Bari Aldo Moro, Bari, Italy; 2 Dominick P. Purpura Department of Neuroscience, Albert Einstein College of Medicine, 840 Kennedy Center, Bronx, NY USA; 3 Department of Bioscience, Biotechnologies and Biopharmaceutic, University of Bari “Aldo Moro”, Bari, Italy

**Keywords:** Aquaporin-4, Astrocyte endfoot, Blood brain barrier, Orthogonal arrays of particles, OAPs,Translational readthrough, Neuromyelitis optica

## Abstract

Brain water homeostasis is essential for the appropriate control of neuronal activity. Furthermore, the encasement of the central nervous system (CNS) by a hard structure, greatly limits its tolerance for the volume changes occurring with acute brain edema, which quickly leads to severe damage or death.

The recent discovery of the extended isoform of AQP4 (AQP4ex), generated by translational readthrough, revealed a potential new mechanism of water transport regulation and polarization at the blood-brain-barrier level.

In the present study we used CRISPR/Cas9 technology to generate an AQP4ex^−/−^ mouse model and evaluate the effect on the overall AQP4 expression, polarization, supramolecular organization in orthogonal arrays of particles (OAPs) and neuromyelitis optica (NMO-IgG) autoantibodies binding.

AQP4ex removal did not cause a decrease in total AQP4 protein expression but completely suppressed the specific location of AQP4 at the astrocyte endfeet. Without AQP4ex, AQP4 was mislocalized and α-syntrophin expression, the selective partner for AQP4 localization, was partially altered. The supramolecular organization of AQP4 in OAPs was subtly altered. Indeed, the absence of AQP4ex reduced the size of AQP4-OAPs but the number of AQP4-OAP pools remained largely the same. More importantly, AQP4ex resulted critical for the binding of pathogenic human NMO-IgG autoantibodies to the brain. Indeed, the absence of AQP4ex completely abolished the binding of NMO-IgG at the perivascular astrocyte endfeet.

This study provides the first direct evidence *in vivo* on the specific role of AQP4ex in AQP4 perivascular OAPs assembly and confinement and reveals AQP4ex as new and important player in neuromyelitis optica.

## Introduction

AQP4 plays a central role in the preservation of central nervous system (CNS) water homeostasis, which is essential for the maintenance of osmotic composition and volume within the interstitial, glial and neuronal compartments [23, 31]. Indeed, even small changes in osmolarity or volume can dramatically alter neuronal activity and circuitry.

Numerous studies have reported altered AQP4 expression in pathological states such as brain ischemia, tumors and neurodegenerative and neuroinflammatory diseases [36]. The common trait of these diseases is the alteration of the physiological features of the astrocytes, a glial cell type that has a crucial role in the homeostasis of the extracellular space of the CNS and in the integrity of various brain interfaces including the blood brain barrier (BBB).

AQP4 is strongly expressed and confined at the astrocyte endfoot covering the BBB [11, 30]. Differently to other AQPs, AQP4 is expressed in several isoforms: the most abundant are M23 and M1 in a ratio of approximately 3:1. These two isoforms are expressed in the form of heterotetramers which further aggregate into the plasma membrane to form well ordered two dimensional supramolecular structures called orthogonal array of particles (OAPs) [19, 25]. OAPs are another exclusive feature of AQP4 [15], the physiological role of which is yet to be resolved.

Recent computational studies have suggested AQP4 as a strong translational readthrough candidate [21]. This mechanism allows the translation of AQP4 mRNAs that can continue beyond the canonical stop codon to a downstream stop codon, generating a C-terminal polypeptide extension. In general, translational readthrough plays important regulatory roles and is functionally important as demonstrated for vascular endothelial growth factor VEGF-Ax [10] and for the large myelin protein zero (L-MPZ) [37]. It has recently been proved that AQP4 mRNA undergoes robust translational readthrough in human and rat tissues to generate AQP4ex, containing a 29-amino-acid C-terminal extension [8]. In vitro experiments have suggested that AQP4ex may modulate supramolecular organization and may regulate water channel activity [8].

Previous studies on the role of AQP4 largely used AQP4-null mouse providing fundamental answers on the role of AQP4, especially in pathological conditions of the CNS [36]. However, this global AQP4 null animal model provided limited information on the physiological role of AQP4 in the CNS and the contribution of the single isoforms could be not addressed.

In particular, the role of OAPs could be addressed only with use of transfected cells with the M1 and M23 isoforms [27, 34] and partially with the use of M23 adenovirus infection in AQP4-KO mouse [35]. These models suggested that the assembly of AQP4 into large OAP complexes could enhance plasma membrane stability to AQP4 water channels, which need to be confined in microdomains not delimited by tight junctions such as the glial processes at the level of the blood–brain barrier (BBB) and determine, in these specific locations, high-polarized water permeability [28]. Whether OAP formation is sufficient to anchor the protein to specific plasma membrane microdomains or whether AQP4 needs to interact with protein complexes such as the dystrophin-glycoprotein complex (DGC) [14] or the actin cytoskeleton to be confined [29], is still unclear. α-syntrophin has been indicated as a privileged partner for AQP4 anchoring in brain astrocyte perivascular processes [3, 24] and fast-twitch skeletal muscle fiber sarcolemma [1, 13]. However, a direct interaction of α-syntrophin with AQP4 has never been demonstrated.

Another interesting and unclear issue for a therapeutically purpose concerns the conformational epitope recognized by the autoantibodies (NMO-IgG) produced by neuromyelitis optica patients that specifically binds to AQP4 [20]. Previous studies have suggested that the conformational epitopes recognized by NMO-IgG are generated when the heterotetramers aggregate in OAPs, since the M1 isoform, unable to form OAPs in transfected cells, show low or no binding of NMO-IgG [17, 27, 32]. Other studies, using engineered monoclonal NMO antibodies have reported similar binding in M1 and M23 transfected cells [6], although a different binding affinity was later reported [7].

In the present study we have used CRISPR/Cas9 technology for the first time to generate an AQP4ex^−/−^ mouse model and evaluate the effect on the overall AQP4 expression, polarization and OAPs distribution.

The results demonstrate a critical role of AQP4ex in the correct targeting of water channels at the BBB interface and provide a new and unexpected view in the organization of OAPs in the CNS and open a promising path for long-lasting unresolved questions and for pharmacological intervention in brain edema and NMO.

## Material and methods

### Generation of AQP4ex-KO mice

The generation of AQP4ex-KO mice was accomplished by Cyagen Biosciences Inc. (Santa Clara, USA) using the new CRISPR/Cas9 technology [18]. The strategy used to prevent the translation of the AQP4ex isoform consisted of modifying the AQP4 stop codon UGA in the codon UAA [21] and introducing two additional stop codons (UAG,UGA). Although in the target sequence two sites for Cas9 activity were identified (the ATTGTCTTCCGTATGACTAGAGG gRNA1 sequence and the AGTGCTGTCCTCTAGTCATACGG gRNA2 sequence), the gRNA1 sequece was selected since it had the highest quality score (84), and a low probability off target sites (highest score 0.9), indicating high specificity. Cas9 mRNA, gRNA generated by in vitro transcription and donor oligo (with targeting sequence, flanked by 120 bp homologous sequences combined on both sides) were co-injected into fertilized eggs for AQP4ex-KO mouse production. The pups were genotyped by PCR, followed by sequence analysis and the positive founders were bred to the next generation. Specifically, founders were crossed with WT animals to generate F1 heteorozygous mice. These mice were then analyzed for the six most probable off-target mutations for the selected gRNA that were identified on the X chromosome, on chromosomes 10, 3 and 4. PCR amplification and sequence analysis confirmed that no off target mutations were introduced at those sites. Finally, F1 heterozygous mice were bred to obtain AQP4ex-KO mice. The pups were genotyped by PCR followed by sequence analysis. A 0.5 mm tail biopsy was cut off and DNA was extracted using a commercial kit (Thermo Scientific Phire Animal Tissue Direct PCR Kit, Thermo Fisher Scientific, USA). The extracted DNA was amplified by PCR using specific primers: Mouse Aqp4-F 5′-GTTGGACCAATCATGGGCGCT-3′ and Mouse Aqp4-R 5′-CCAGCTTCCTCACAGAGGTGTCCA-3′. The 617 bp long PCR products were purified using a QIAEX II Gel Extraction Kit (QIAGEN, Germany) and sequenced by an external facility (BMR Genomics, Padova, Italy). The amplified DNA was also digested using Mae III restriction enzyme (Mae III from Methanococcusaeolicus PL-15/H, Roche, Switzerland) to obtain three knockin (125 bp, 220 bp, 272 bp), two wild-type (226 bp and 391 bp) and five heterozygous (125, 220, 226, 272 and 391 bp) DNA fragments.

### Animals

Experiments were conducted in accordance with the European directive on animal use for research and the project was approved by the Institutional Committee on Animal Research and Ethics of the University of Bari and by the Italian Health Department (Project n° 189/2017-PR and Project n° 2A298.N.2G1). Mice were kept under a 12 h dark to light cycle, constant room temperature and humidity (22 ± 2 °C, 75%), with food and water ad libitum. Experiments were carried out on mice of different ages from 2 to 12 months-old. Wild type and AQP4ex-KO mice were anesthetized by intraperitoneal injection of Ketamine (100 mg/kg body weight). All experiments were designed to minimize the number of animals used and their suffering.

### Antibodies

The following primary antibodies were used: rabbit polyclonal anti-AQP4 (Sigma, Saint Louis, Missouri, USA) diluted to 1:2000 for immunoblot analysis and 1:1000 for immunofluorescence; rabbit polyclonal anti-α1-Syntrophin (Sigma, Saint Louis, Missouri, USA) at 1:1000 for immunofluorescence experiments; mouse monoclonal anti-human Dystrophin (Monosan, PB Uden, The Netherlands) used at 1:500 for immunofluorescence; mouse monoclonal anti-CD31 (Dako, Santa Clara, California, USA) diluited to 1:100 for immunofluorescence experiments; Custom rabbit polycolonal anti mouse AQP4ex generated against the peptide DSTEGRRDSLDLASC within the mouse AQP4 carboxy-extension (GenScript) and diluted 1:5000 for immunoblotting and 1:4000 for immunofluorescence. For immunofluorescence, AlexaFluor 488 anti-rabbit, AlexaFluor 488 anti-human, AlexaFluor 488 anti-mouse and AlexaFluor 594 anti-mouse were used (all from Life Technologies, Thermo Fisher Scientific, Carlsbad, California, USA) at a dilution of 1:1000; for immunoblotting, anti-rabbit IgG-HRP (Bio-Rad, California, USA) was used at 1:3000.

### NMO sera

Serum from 14 patients with Neuromyelitis Optica was used for this study. Sero-negative patients were used as negative controls. All the sera were tested for AQP4 antibodies (NMO-IgG) using both an in-house CBA method [32] and a commercial kit (Euroimmune, Lubeck, Germany) at the Laboratory of Neurochemistry of the Department of Basic Medical Sciences, Neuroscience and Sense Organs, University of Bari.

### Immunofluorescence on tissue sections

Immunofluorescence experiments were performed as previously described [27]. Briefly, isolated tissues were fixed in 4% PFA solution at 4 °C overnight and then, after washing for 1 h in PBS, were immersed in 30% sucrose solution in PBS overnight. After washing in PBS, tissues were embedded in Tissue-Tek OCT compound (Sakura, The Netherlands) and frozen at − 80 °C. After blocking, sections were incubated with primary antibodies for 1 h at room temperature in blocking solution (0.1% Gelatin in PBS), washed for 30 min and then incubated with secondary antibodies for 1 h. Finally, the sections were washed for 15 min in PBS and mounted in PBS-glycerol (1:1) pH 8.0, containing 1% n-propyl gallate.

In the case of NMO-IgG staining, isolated tissues were frozen without fixation. Sections of 8 μm thickness were cut on a cryostat (CM 1900; Leica) at − 20 °C and stored on poly-l-lysine glass slides (positively charged) at − 80 °C. In this case, tissue sections were fixed at the end of the staining procedure to preserve conformational epitope integrity.

Finally, sections were viewed with a Leica DM2500 LED fluorescence microscope using 20X/0.55 and 40X/0.80 PL FLUOTAR objectives and photographed with a Leica DFC7000 T CCD camera. Confocal images were obtained using an automated inverted Leica TCS SP8 confocal microscope with a 100X HC PL Apo oil CS2 objective.

### Sample preparation for SDS-PAGE

For SDS-PAGE [25], harvested tissues were frozen in liquid nitrogen and stored at − 80 °C. Proteins were extracted from stored explanted brain in 5–7 volumes of RIPA buffer (10 mM Tris-HCl, pH 7.4; 140 mM NaCl; 1% Triton X-100; 1% Na^+^deoxycholate; 0.1% SDS; 1 mM Na_3_VO_4_; 1 mM NaF and 1 mM EDTA) added with a cocktail of protease inhibitors (Roche, Milan, Italy). The lysis was performed on ice for 1 h and the samples were then centrifuged at 21,000×g for 1 h at 4 °C. The supernatant was collected and the proteins were measured with a bicinchoninic acid (BCA) Protein Assay Kit (Thermo Scientific, Waltham, Massachusetts).

### SDS-PAGE and Western blot analysis

The electrophoresis and immunoblotting were performed as previously described [27]. Briefly, proteins were separated on 13% SDS/PAGE and transferred to polyvinylidenedifluoride (PVDF) membranes (Millipore, Burlington, Massachusetts, USA) for immunoblot analysis. Membranes were incubated with primary antibodies overnight, washed, and incubated with peroxidase-conjugated secondary antibodies at room temperature for 45 min. Reactive proteins were revealed using an enhanced chemiluminescent detection system (Clarity Western ECL Substrate, Bio-Rad, California, USA) and visualized on a Chemidoc Touch imaging system (Bio-Rad, California, USA). Densitometry analysis was performed using Image Lab (Bio-Rad, California, USA).

### Sample preparation for BN-PAGE

Samples for BN-PAGE were prepared as described previously [25]. Briefly, tissues were dissolved in seven volumes of BN buffer (1% Triton X-100, 12 mM NaCl, 500 mM 6-aminohexanoic acid, 20 mM Bis-Tris, pH 7.0, 2 mM EDTA, 10% glycerol) plus protease inhibitor cocktail (Roche, Basel, Switzerland). Tissues were lysed on ice for 1 h, and the samples were centrifuged at 17,000 x g for 30 min at 4 °C. The supernatants were collected, and the total protein content was calculated using the BCA Protein Assay Kit (Thermo Scientific, Waltham, Massachusetts). Thirty micrograms of protein sample was mixed with 2 μl of Loading Buffer (5% of Coomassie Blue G-250, 750 mM aminocaproic acid) and 10% Glycerol by volume.

### BN-page

BN-PAGE was performed as previously reported [25]. Briefly, polyacrylamide native gradient gels (3–9%) were prepared in Mini-PROTEAN (Bio-Rad, California, USA) with 1.5 mm spacers. Protein samples, prepared as previously described, were loaded in each lane. Anode buffer (25 mM Imidazole pH 7.0) and Cathode Buffer Blue (50 mM Tricine, 7.5 mM Imidazole, 0.02% Coomassie Blue G-250) were used as running buffers and the electrophoresis was performed at 6 mA at 4 °C. After the tracking line of Coomassie Blue G-250 dye reached half of the gel, cathode buffer blue was removed and substituted by cathode buffer (50 mM Tricine, 7.5 mM Imidazole). The electrophoresis continued at 6 mA at 4 °C and was stopped when the blue line had left the bottom of the gel. Finally, proteins were transferred to PVDF membranes (Millipore, Burlington, Massachusetts, USA) for immunoblot analysis, as described below.

### Immunoprecipitation

Protein lysates were prepared as described previously [27] in Lysis Buffer (1% Triton X-100, 10 mM Tris-HCl, pH 7.4, 150 mMNaCl, 1 mM EDTA, 1 mM ethylene glycol tetraacetic acid [EGTA]) and were incubated overnight at 4 °C on a mechanical rotator with anti-AQP4ex antibody, anti-AQP4 antibody or with NMO sera. The next day, 50 μL of prewashed agarose beads (rProteinGAgarose; Invitrogen, Carlsbad, California, USA) was added and incubated for 1 h at 4 °C. Negative controls were set up by omitting antibodies. To precipitate the immuno complexes, the samples were centrifuged at 17,000 x g at 4 °C for 5 min and washed five times with washing buffer (0.2% Triton X-100, 10 mM Tris-HCl, pH 7.4, 150 mM NaCl, 1 mM EDTA, 1 mM ethylene glycol tetraacetic acid [EGTA]) plus Protease Inhibitor Cocktail. Proteins were eluted from the agarose beads by adding 60 μL of Laemmli Sample Buffer 2X (Bio-Rad, California, USA) and incubating at 37 °C for 10 min. The eluted proteins were subjected to SDS-PAGE as previously described.

### Water transport measurements

Cell-volume changes in primary cultured astrocytes from AQP4ex null, AQP4 KO and WT mice were analyzed by calcein-quenching fluorescence assay [22]. Astrocyte primary cultures were prepared as previously described [26]. Cells were seeded on black, clear bottom microplates (Corning, New York, USA) at a density of 20,000 cells per well and used 24 h after plating at which time they were 80–85% confluent, as previously described [22]. Fluorescence signal changes in calcein-loaded glial cells after hypotonic or hypertonic gradient were recorded on a Flex Station3 plate reader (Molecular Devices, San Jose, USA) equipped with an integrated automatic liquid handling module. The osmotic challenge was applied 15 s after the beginning of the data acquisition by adding an appropriate volume of hypo- (NaCl-free DPBS) or hypertonic (D-mannitol 0.5 M) solution in order to obtain 240 mOsm/L or 360 mOsm/L final extracellular osmolarity, respectively. The changes in fluorescence intensity were directly proportional to changes in cell volume. The signal decreased upon addition of hypertonic solution as a consequence of water efflux and cell shrinkage. The fluorescence signal increased with the hypotonic solution due to cell swelling. Data acquisition was performed using SoftMax Pro software, and the data were analyzed with Prism (Graph Pad) software. The time constant of cell volume variation was obtained by fitting the data to an exponential function.

### Statistical analysis

Mean ± standard error is reported in the results. Statistical analysis was performed using GraphPad Prism 5 (GraphPad, San Diego, USA) by t-test for unpaired data or Analysis of variance (ANOVA) followed by Tukey’s test. A *P* value < 0.05 was considered statistically significant.

## Results

### Generation of AQP4_ex_-KO mice

**Fig. 1:**
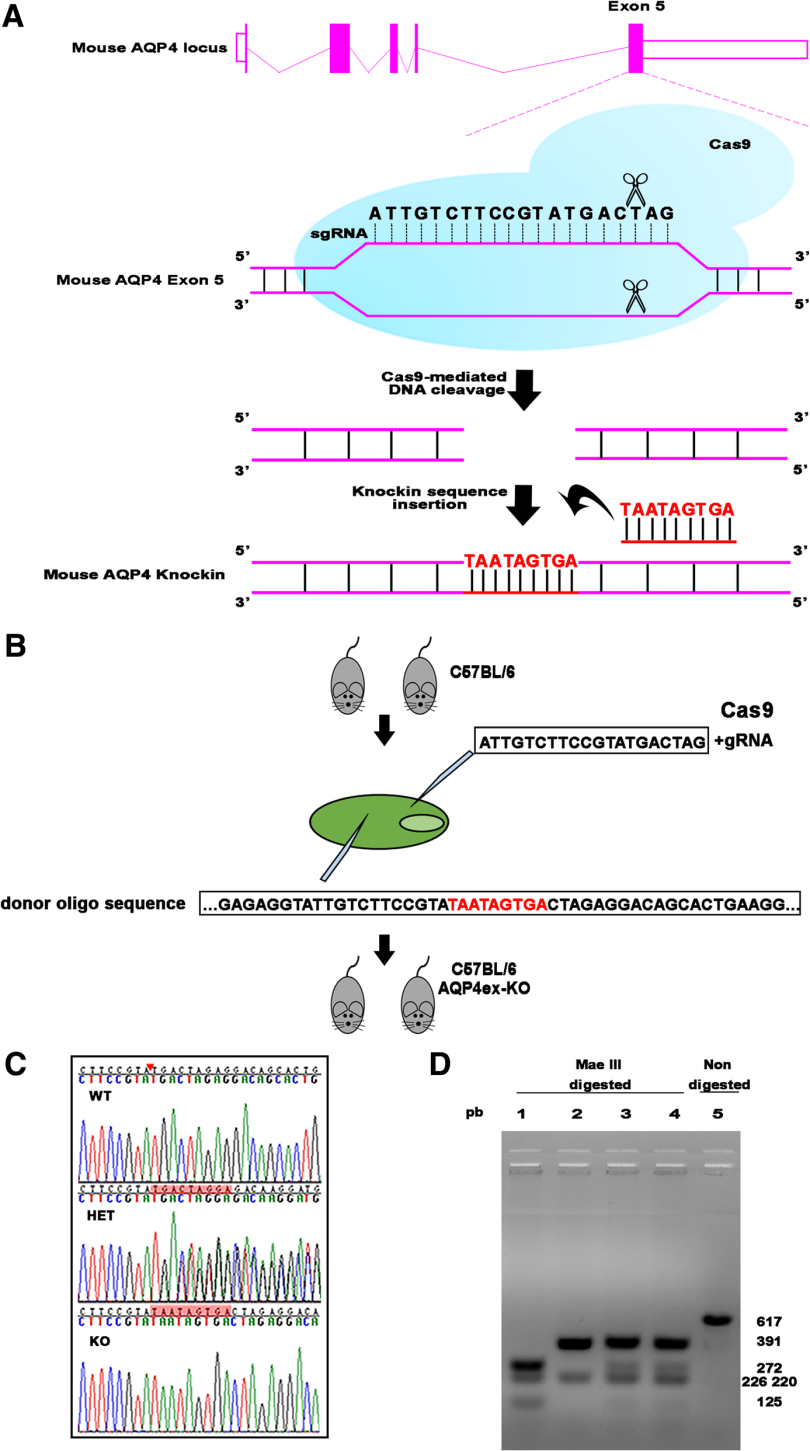
Generation of the AQP4ex −/− mouse. **a** Schematic depiction of the targeting strategy. The mouse Aqp4 gene contains five exons (vertical bars). Exon 5 contains the target site for the knockin TAATAGTGA sequence. After Cas9-mediated DNA cleavage, the knockin TAATAGTGA sequence was introduced into Exon 5 by homology-directed repair. **b** Mouse AQP4ex-KO generation. gRNA obtained by in vitro transcription and donor oligo were co-injected into fertilized eggs for KI mouse production. **c** DNA sequencing analysis. The 617 bp long PCR product was purified and sequenced to analyze the DNA sequence in the target site (highlighted in red) of wild-type (WT) heterozygous (HET) and KO mice. **d**. DNA restriction enzyme analysis. Mae III fragments obtained by complete digestion of KO, lane 1 (125, 220, 272 bp) wildtype, line 2 (226 bp and 391 bp) and heterozygous, line 3 and 4 (125, 220, 226, 272 and 391 bp) PCR products. The PCR product without MAE III enzyme digestion is shown in line 5

AQP4ex deficient mice were designed for the selective absence of the extended isoform of aquaporin-4. CRISPR/Cas9 technology was used for this purpose (Fig. 1). The abolishment of the translation of AQP4ex isoform was obtained by changing the “weak” stop codon UGA at position 969 with the “strong” codon UAA according to a previous study [21] and by adding two successive stop codons (Fig. 1a-c).

Sequence analysis of the 617 bp long PCR product of the tail extracted DNA from wild type, heterozygous and AQP4ex-null mice obtained from the same breeding confirmed the correct insertion of the mutation in the genome (Fig. 1c).

The inserted mutation generated a new Mae III restriction site that was used for the animal screening after the PCR amplification (Fig. 1d). The analysis of 65 live births from AQP4 +/− mating showed 15 (+/+), 35 (+/−), 15 (−/−) genotypes, a distribution that did not differ significantly from the Mendelian 1:2:1 ratio. Male and female phenotype proportion was similar and AQP4ex-KO mice bred normally, with no evidence of impaired fertility and did not show any visible sign of suffering phenotype.

### AQP4ex expression and localization in the mouse CNS

**Fig. 2:**
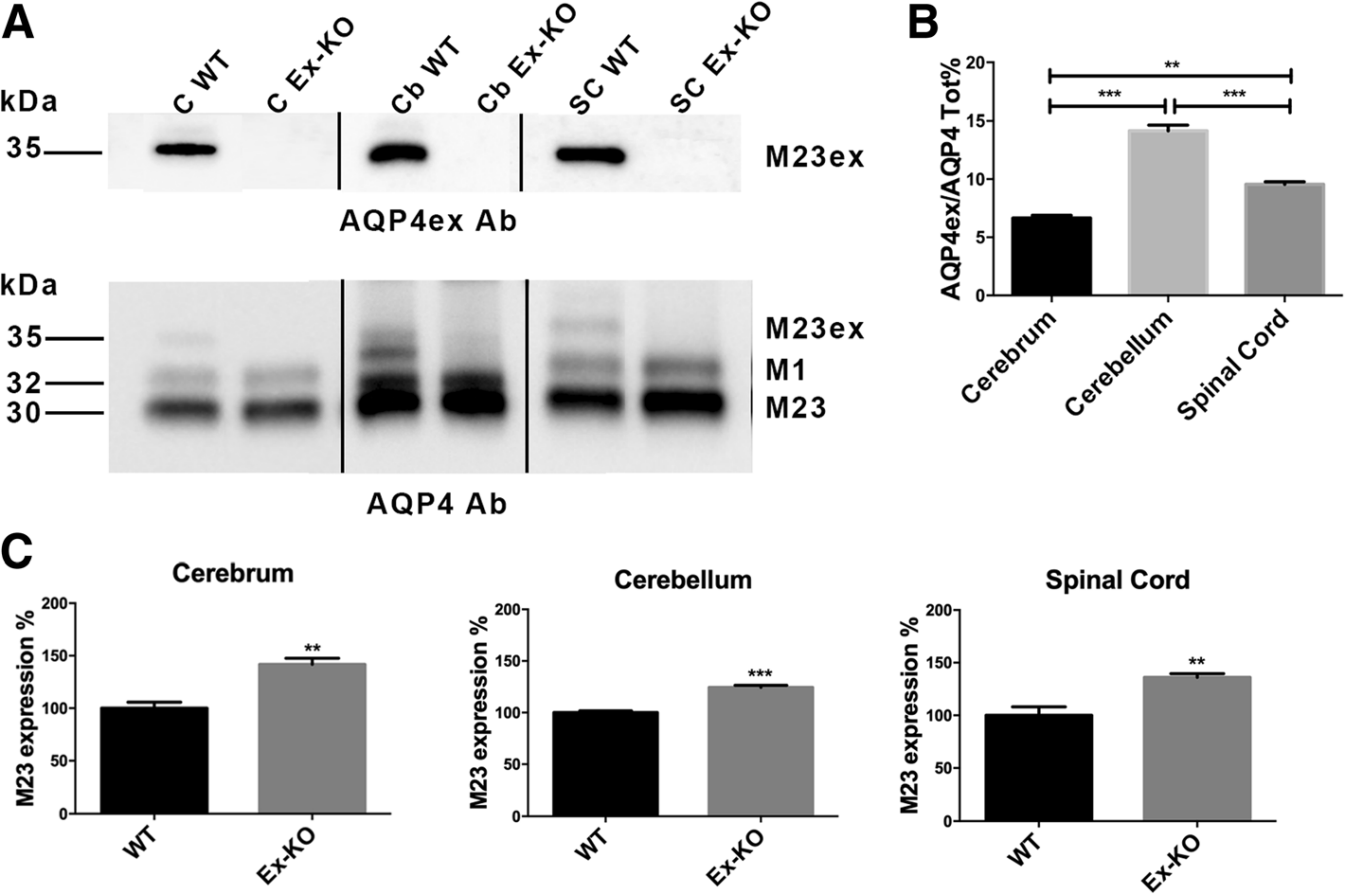
Immunoblot analysis of AQP4 isoforms in WT and AQP4ex-KO mice CNS. **a** A typical immunoblot is shown with cerebrum (C), cerebellum (Cb) and spinal cord lysates (SC) probed with anti-AQP4ex (top) and anti-AQP4 antibodies (bottom). Note the absence of the M23ex isoform of about ~ 35 kDa in the AQP4ex-KO extracts. Using commercial AQP4 antibody (bottom), three bands of ~ 30, ~ 32 and ~ 35 kDa were detected corresponding to AQP4-M23, AQP4-M1 and AQP4-M23ex, respectively. **b** Bar chart showing the mean ± SE of the percentage of AQP4ex relative to the total AQP4 measured by immunoblotting in the CNS tissues of WT mouse (****p < 0.0001; ** *p* < 0.001, *n* = 5). **c** Bar chart showing the mean ± SE of expression levels of AQP4-M23 isoform in WT and AQP4ex-KO cerebrum, cerebellum and spinal cord. Note that the amount of the canonical M23-AQP4 increases in the CNS of AQP4ex-KO mice (Student’s t-test ***p < 0.001; ** *p* < 0.01, *n* = 3)

**Fig. 3:**
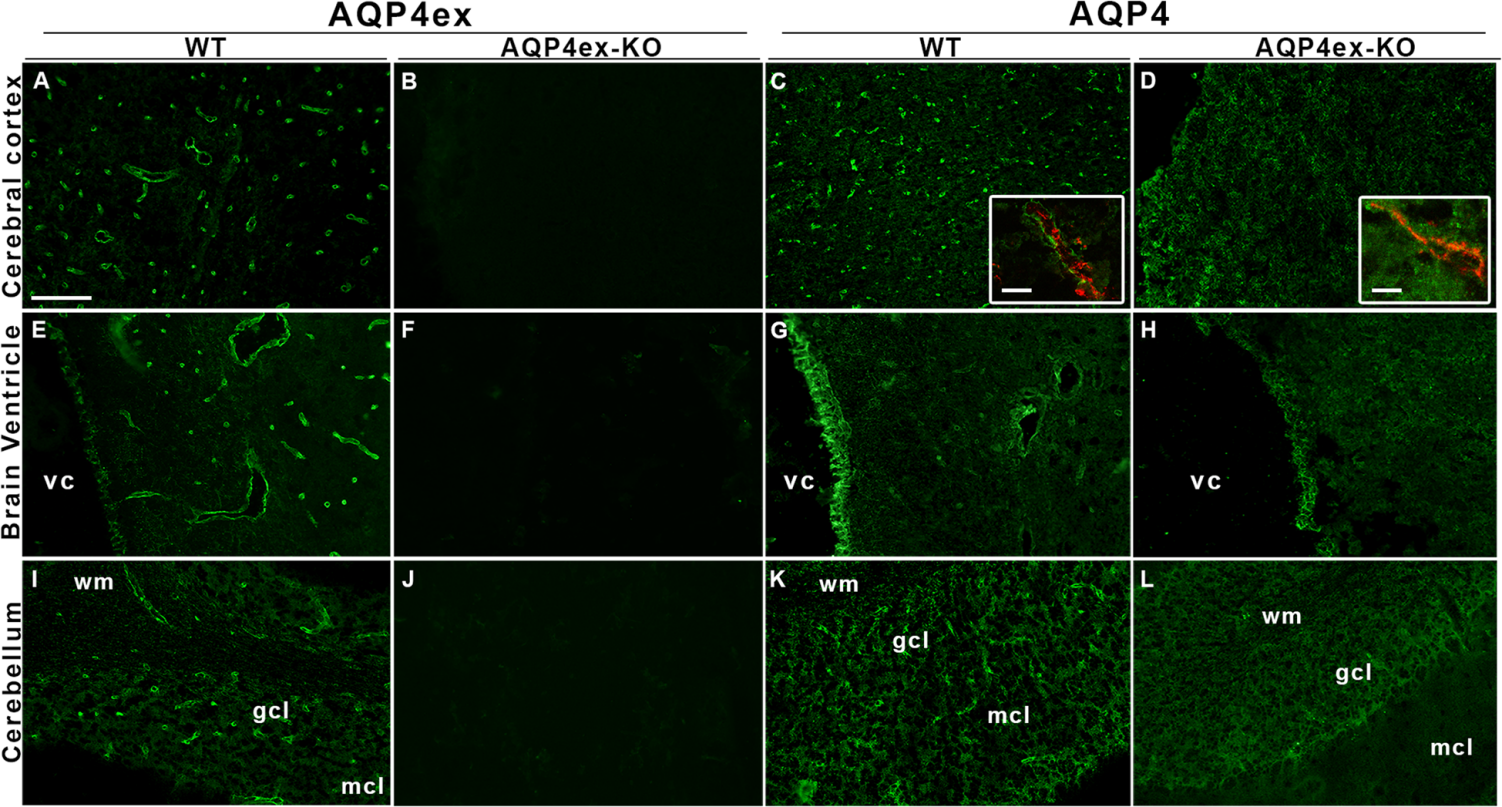
Immunolocalization of AQP4 isoforms in WT and AQP4ex-KO mouse brain. The cerebral cortex and the cerebebellum (granular, gcl and molecular cell layer, mcl) are shown together with the inner region (choroid plexus) containing the 4th ventricle cavity (vc) stained with AQP4ex and AQP4 global antibodies (**a**, **e**, **i**). Perivascular staining of AQP4ex is shown in WT mouse (**a**, **e** and **i**), while the signal is absent in KO tissues (**b**, **f**, **j**). The inserts (panel **c** and **d**) show a magnified view, obtained by high resolution confocal microscopy, of the perivascular area in WT and KO cerebrum with vessels stained by CD31 antibody (red staining). Note the increased staining of AQP4 on the astrocyte membrane facing the neuropil in the AQP4ex-KO cerebrum. Scale bar 5 μm. A faint staining of AQP4ex was also observed in ependymal cells lining the ventricular cavity (**e**) and the dense glial processes of the gcl (**i**) in WT mouse. In AQP4ex mice the staining of AQP4 appeared still present although reduced (**h**,**l**). WM, white matter. Scale bar 50 μm

To evaluate the consequences of the absence of AQP4ex on the overall expression of AQP4 (ie M1 and M23 isoforms) in the CNS, immunoblot (Fig. 2) and immunofluorescence experiments (Fig. 3) were performed. A peptide (DRTESRQDSLELSS) specific antibody that exclusively recognizes the mouse AQP4ex isoform was produced for this purpose. AQP4ex probed immunoblots (Fig. 2a) of CNS extracts revealed a ~ 35 kDa band in wild type mouse tissues, corresponding to AQP4-M23ex, but not in the AQP4ex knockout mouse tissues. This result was confirmed by probing the same immunoblot membrane with the antibody that recognizes all the AQP4 isoforms. At higher exposure the M1ex isoform (~ 38 KDa) could also be detected by immunoblot in WT but absent in KI animals. These results demonstrate that the stop codon knock-in strategy used to generate AQP4ex Knockout mice was successful.

Densitometric analysis showed that the extended isoforms, generated by readthrough in the CNS of mouse, constituted approximately 10% of all AQP4 isoforms with the highest expression in the cerebellum (Fig. 2b). As expected, the amount of the canonical AQP4 isoform M23 increased in the CNS of AQP4ex-KO mice (Fig. 2c) indicating that the introduced stop codons tighly work.

Cryosections of cerebrum and cerebellum from WT mice stained with anti-AQP4ex antibodies confirmed a strong expression of the extended isoform in these tissues (Fig. 3). In particular, AQP4ex showed a polarized distribution in the cerebral cortex mostly confined to the pericapillary astrocyte endfeet (Fig. 3a and e). Moreover, AQP4ex expression was also detected, although to a lesser extent, at the basolateral membrane of the ependymal cells and also a faint signal at the glia limitans interna in the periventricular region (Fig. 3e). The immunofluorescence signal of AQP4ex in AQP4ex-KO mice was completely abolished in all the areas where AQP4ex is expressed. Surprisingly, the use of the global anti-AQP4 antibody revealed the complete disappearance of the perivascular glial processes staining in AQP4ex-KO mouse and a strong and dense reticular-like signal of the canonical isoforms in the cerebral cortex (Fig. 3d and h).

AQP4ex localization was also investigated in the cerebellum, in which dystrophin-dependent and independent pools of AQP4 have been described [25]. AQP4ex was mainly localized at the perivascular astrocyte processes in the granule cell layer (gcl), although a significant staining was also detected in the dense network of astrocyte processes of the gcl (Fig. 3i). In AQP4ex-KO animals, the perivascular AQP4 staining of the gcl was lost while the staining of the scattered processes was still abundantly present and appeared only slightly reduced (Fig. 3l). As previously reported [11], AQP4 is also expressed at the basolateral membrane of the ependymal cell layer lining the ventricle cavities of the CNS. AQP4ex was found expressed, although to a lesser extent, at this epithelial layer and in the same plasma membrane location as the canonical AQP4. After AQP4ex suppression, the staining of AQP4 appeared to be only reduced but not absent as occurs for the glial processes near the brain capillaries.

### Effect of AQP4ex suppression on AQP4 aggregation state

**Fig. 4:**
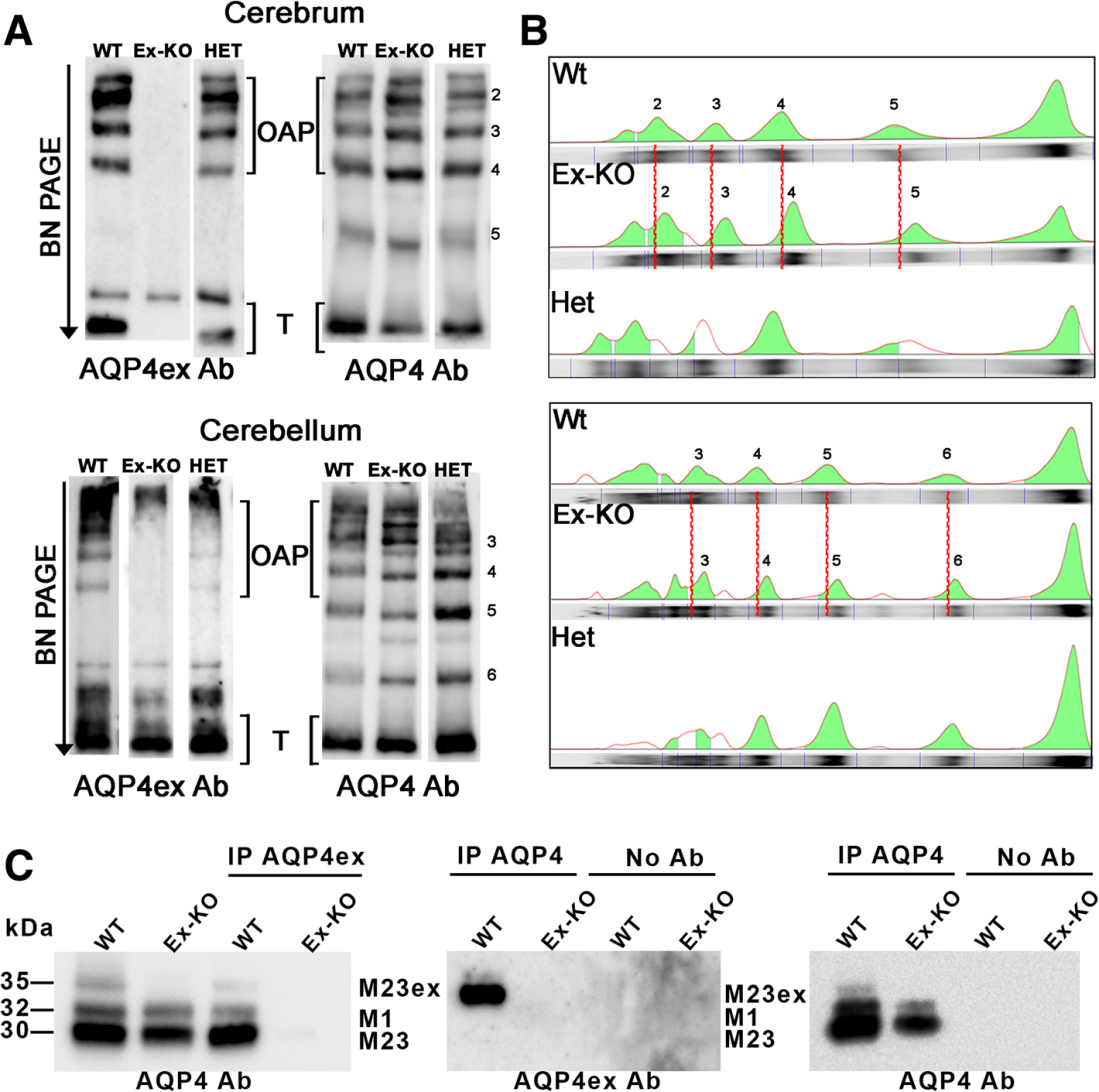
AQP4 and AQP4ex supramolecular organization in mouse brain. **a** BN-PAGE analysis of AQP4 and AQP4ex expression and supramolecular aggregation in the cerebrum and cerebellum of wild-type (WT), heterozygous (HET) and AQP4ex-KO (Ex-KO) mice. The high molecular weight of AQP4 pools (OAP) and the tetrameric form of AQP4 (T) are indicated. **b** Four different pools of AQP4 were analyzed for the band shift analysis. The profile of the cerebrum AQP4 pools (top) and the cerebellum AQP4 pools (bottom) for the three animals is shown and the red dotted lines represent the shift of the analyzed pools for the cerebrum (2–5) and the cerebellum (3–6). **c** AQP4 isoform interaction by IP experiment analysis. Brain homogenates were immunoprecipitated with anti-AQP4ex (IP AQP4ex) and anti-AQP4 (IP AQP4) antibody and subjected to immunoblot analysis with anti-AQP4 (left) and anti-AQP4ex (middle) antibodies, respectively. Samples immunoprecipitated with anti-AQP4 antibody were probed for AQP4 as control (right). The top band at 35 kDa corresponds to AQP4ex, and the bottom bands at 30 and 32 kDa correspond to the canonical AQP4 isoforms. The first two lanes of the immunoblot of the left panel contain brain lysates probed with AQP4 antibodies loaded as positive controls

BN-PAGE is a biochemical technique largely used for the analysis of AQP4-supramoleclar organization [25]. BN-PAGE experiments were performed in order to evaluate the effect of AQP4ex absence on OAP composition. Figure 4 shows a typical experiment with CNS samples from WT, HE and KO animals. Immunoblots performed using AQP4ex antibodies revealed the expression of AQP4ex in large size AQP4 pools in WT and heterozygous animals, which were absent in KO animals. In addition, when the same samples were analyzed with the global AQP4 antibody, it was found that the absence of AQP4ex determined a reduction in the size of AQP4-WT pools, while the number of AQP4 pools was not altered. This suggests that AQP4ex interacts, *in vivo*, with the canonical AQP4 isoform in each pool and regulates the size of AQP4 pools.

To evaluate the interaction between AQP4ex with the canonical AQP4 isoforms (M1 and M23) immmunoprecipitation (IP), experiments were conducted with WT and AQP4ex-KO brain lysates. IP using AQP4ex antibodies allowed the pull-down of AQP4ex (M23ex), only in WT extracts, while the IP using AQP4 antibodies also revealed the pull-down of AQP4ex only in WT extract. These data corroborate the interaction between AQP4ex with the other AQP4 isoforms in naturally expressing tissues.

### Effect of AQP4ex suppression on astrocyte osmotic water permeability

To evaluate the effect of AQP4ex suppression on water transport properties, primary cultures of AQP4ex null astrocytes were prepared and their osmotic behavior was compared to WT and AQP4-KO astrocytes. Astrocytes from AQP4-KO mice were used to evaluate the water transport in the absence of AQP4 water channels and thus to establish a range of measurements between the two extreme conditions: WT (presence of water channels) and AQP4-KO (absence of water channels). A functional assay based upon calcein fluorescence quenching was applied for this purpose [22].

**Fig. 5:**
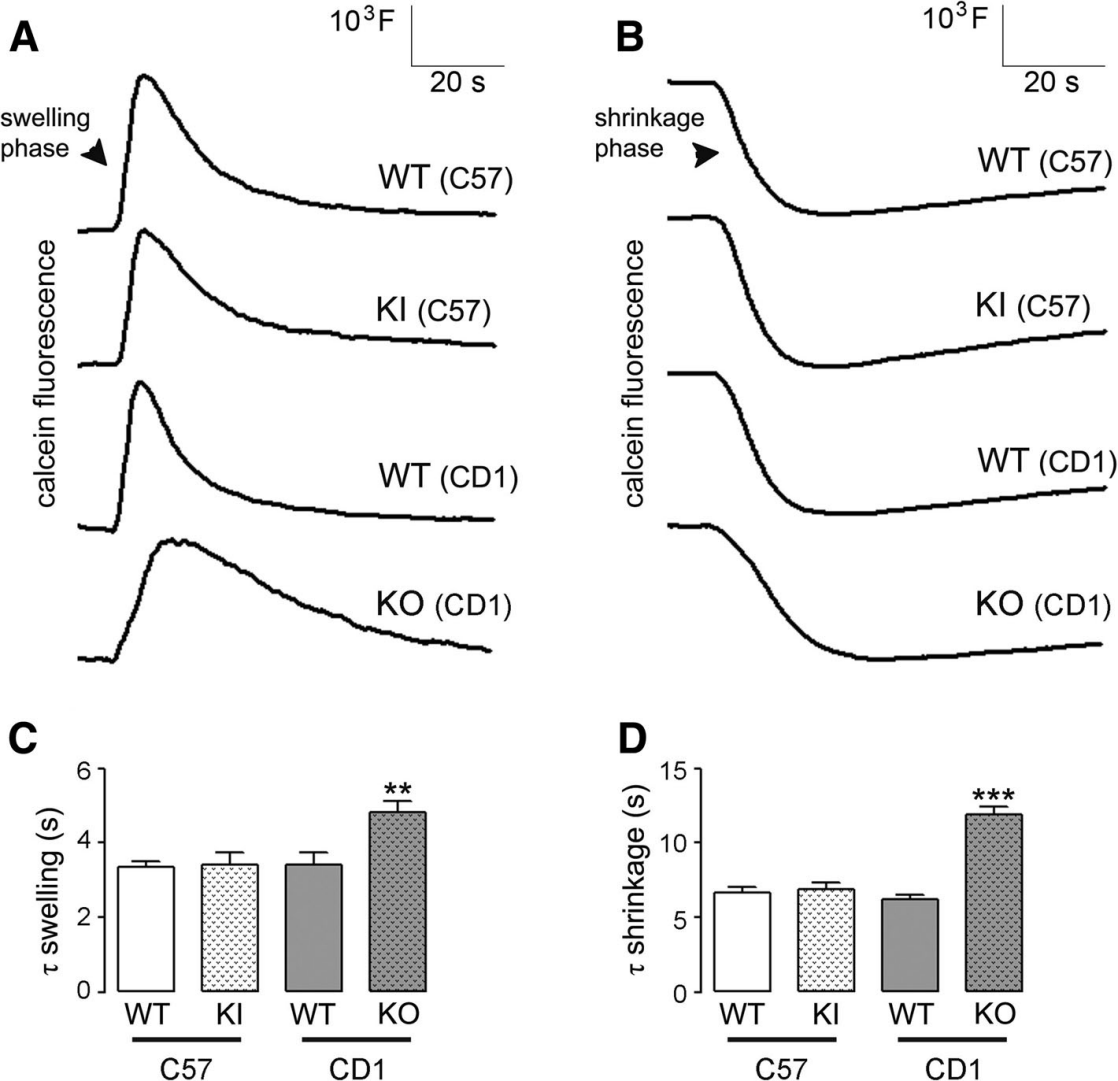
Water transport measurements in astrocytes primary cultures. Calcein-quenching measurement of osmotically induced volume changes in primary cultured astrocytes from AQP4ex null, AQP4-KO and WT mice. **a** Typical traces of cell volume changes, recorded for the indicated astroglial cells, upon exposure to a hypotonic (240 mOsm/L; Δ 60 mOsm/L) (**a**) or hypertonic (360 mOsm/L; Δ 60 mOsm/L) **b** solutions. Note in A the recovery of the fluorescence, as a consequence of the volume regulatory decrease following the hypotonic shock. **c,d**, Histograms showing the mean ± SE values of time constants (τ) obtained by fitting an exponential curve to the cell swelling (**c**) or shrinking (**d**) phases of astrocyes after osmotic challenges obtained from 15 to 22 different measurements of three sets of independent experiments (***p < 0.0001; **p < 0.001)

Figure 5 shows representative data for the time courses of cell swelling (A) and shrinking (B) after hypotonic or hypertonic shocks, respectively, recorded in astrocytes derived from the different mouse strains. The analysis of both swelling and shrinking phases (C,D) revealed no significant differences of time constant values between WT and AQP4ex-KO astrocytes. As expected, the speed of AQP4-KO astrocytes was, instead, significantly slowed compared to WT astrocytes.

### AQP4ex interaction with component of dystrophin complex in the central nervous system

**Fig. 6:**
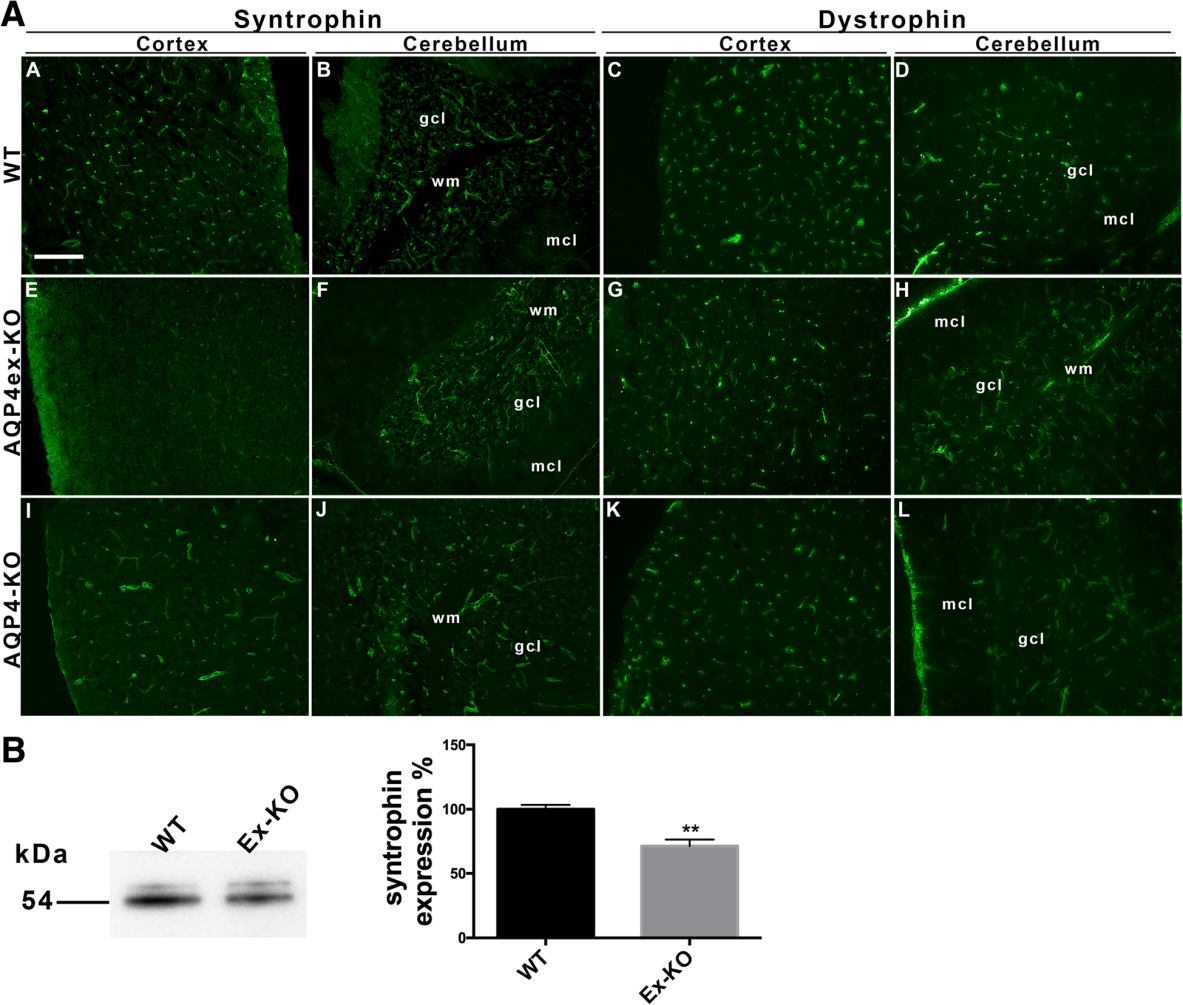
α-syntrophin and dystrophin expression in AQP4ex deficient mice. **a** Expression levels evaluated by IF in WT and AQP4ex –KO mice. For comparison AQP4-KO mice were also analyzed. Note that the perivascular staining of syntrophin in the cortex of AQP4ex-KO mouse is altered (E) while in the cerebellum it appears slightly reduced (F) in both granular (gcl) and molecular cell layers (mcl). No major changes in the dystrophin immunofluorescence signal were found the same areas. wm, white matter. Scale bar 50 μm. **b** Immunoblot analysis (left) of syntrophin expression in WT and AQP4ex-KO mice cerebrum cortex. Left, histogram showing syntrophin protein levels measured by immunblot in cerebrum (mean +/−SE, *p < 0.05; *n* = 6)

It is well known that the absence of an integral dystrophin complex [3, 12, 24] affects AQP4 perivascular expression. Immunofluorescence experiments were performed to evaluate whether the targeted deletion of the extended AQP4 isoforms influences α-syntrophin and dystrophin distributions in the mouse CNS. Surprisingly, the perivascular expression of α-syntrophin was significantly reduced (Fig. 6e) in AQP4ex-KO cerebral cortex, while in the granular cell layer of the cerebellum the perivascular expression of α-syntrophin, was less reduced (Fig. 6f). Differently to that observed for α-syntrophin, dystrophin immunoreactivity of the cerebrum and cerebellum perivascular astrocyte processes (Fig. 6c-d) were not altered (Fig. 6g-h) in AQP4ex-KO mouse. Immunoblot experiments confirmed a reduction of ~ 40% of α-syntrophin in the AQP4ex cerebrum compared to WT cerebrum (Fig. 6b).These results indicate that the interaction between AQP4ex and α-syntrophin is a strong determinant for both being co-localized at the perivascular pole of glial endfeet.

### NMO autoantibodies binding on AQP4ex-KO mice cerebral cortex

**Fig. 7:**
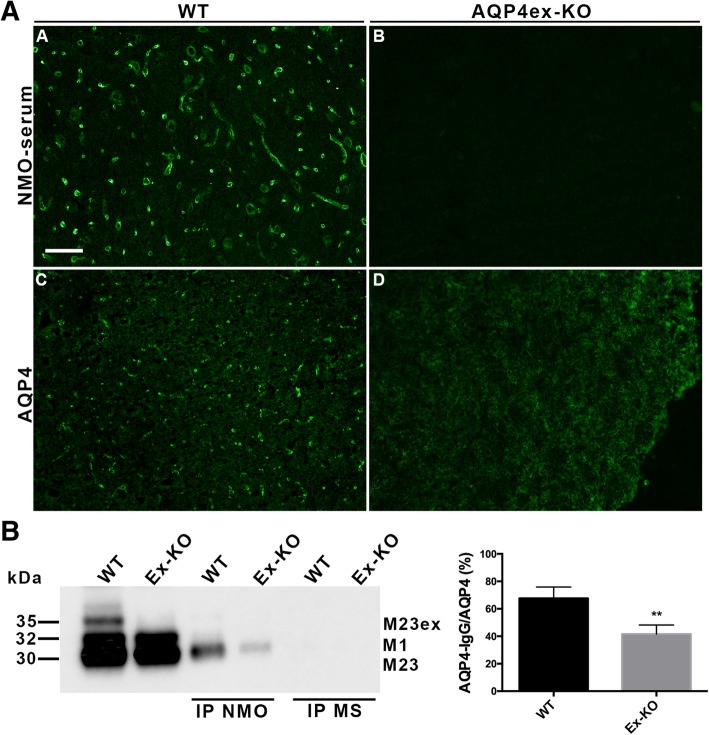
NMO-IgG binding in the brain of AQP4ex-KO mouse. **a**, Immunofluorescence analysis of cerebral cortex of WT and AQP4ex-KO mice stained with NMO-IgG (A,B) and AQP4 (C,D). Note the selective labeling of NMO-IgG to the pericapillary astrocyte processes in WT cortex (A), which is lost in the absence of AQP4ex (B), representative of 14 different NMO-AQP4 positive sera. **b**, Immunoprecipitation of AQP4 isoforms with NMO sera from WT and AQP4ex-KO cerebrum membrane vesicle extracts. Left, immunoblot probed with AQP4 antibodies showing AQP4 isoforms detected in protein vesicle extracts (WT and AQP4ex-KO) in the first two lanes and in the subsequent lanes the IP with NMO serum and MS serum. Right, densitometric analysis of immunoblot results with 3 different NMO sera. Results are shown as the percentage of AQP4 immunoprecipitated by NMO sera in WT and AQP4ex-KO samples to the total AQP4 immunoprecipitated with anti-AQP4 antibody (AQP4). Mean +/− S.E.M., **p < 0.001)

Previous studies have reported NMO autoantibodies (AQP4-IgG) binding preferentially to AQP4-OAPs in transfected cells and in brain; more selectively to those localized to the perivascular region and dystrophin-dependent pool [27, 33]. Thus, immunofluorescence experiments were performed to evaluate the extent of NMO sera binding to AQP4-OAPs in AQP4ex mice. For this purpose, a group of sera from AQP4-IgG positive patients (*n* = 14) were tested on unfixed cryosections of WT and AQP4ex null mice cerebrum. As previously described [27], all NMO sera preferentially recognized the AQP4 dystrophin- dependent perivascular pools in WT animals (Fig. 7a), while the AQP4ex-KO cerebrum showed no detectable perivascular signal (Fig. 7b). IP experiments were then conducted with the NMO sera for a more quantitative evaluation of the AQP4-IgG binding. IP results show about 50% reduction in the AQP4 pull-down capacity with AQP4ex-KO extract compared to WT extract. These data suggest that the reduced binding of AQP4-IgG to AQP4 in AQP4ex-KO cerebrum is linked to the altered organization of AQP4 supra-structures and indicate AQP4ex involved in NMO-IgG binding.

## Discussion

In this study we have used CRISPR/Cas9 technology for the first time to generate an aquaporin knock-in model to evaluate the role of the recently discovered AQP4ex isoform in the CNS.

### Role of AQP4ex in glial endfeet AQP4 expression

As reported in human and rat CNS, mouse AQP4ex is expressed predominantly by glial endfeet surrounding brain capillaries, although significant expression was found in ependyma cells outlining the cerebral ventricles. Importantly, suppressing AQP4ex totally abolished the perivascular staining of AQP4, indicating that AQP4ex expression is fundamental for the functional expression of AQP4 at the blood-brain interface. Furthermore, BN-PAGE and IP experiments demonstrated that AQP4ex interacts with the canonical isoforms and thus it can be concluded that to secure AQP4 at the perivascular pole the presence of AQP4ex is essential.

**Fig. 8:**
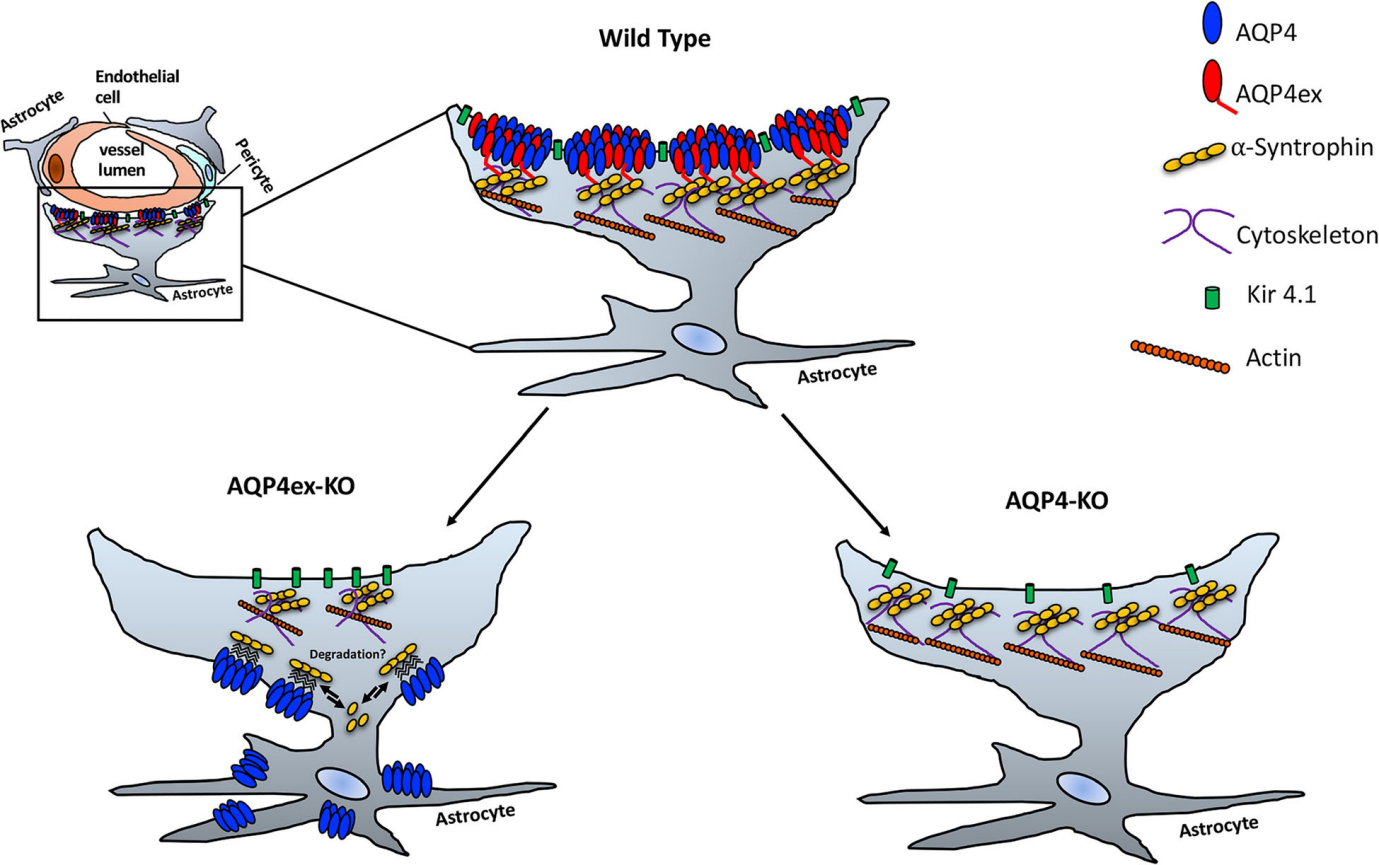
Schematic drawing on the role of AQP4ex at the glial perivascular microdomain

It is amply documented that AQP4 localization depends on α-syntrophin [2] and that the C-terminal extension is required for this interaction [8, 9, 24]. Interestingly, in the brain cortex of AQP4ex-KO animals α-syntrophin expression appears largely affected indicating AQP4ex to be important for the correct α-syntrophin localization. This mutual relationship is a new finding and envisages AQP4 as a novel element for the correct anchoring of proteins at the perivascular pole. Interestingly, dystrophin expression appeared not to be altered at the perivascular pole indicating that although α-syntrophin expression is affected the proteins of the dystrophin complex should be correctly targeted. Since there is no alteration of It is amply documented that AQP4 localization depends on α-syntrophin in AQP4-KO mice, we can conclude that the large misslocalization of AQP4-OAP depleted of AQP4ex more severely affects the expression of α-syntrophin than the absence of AQP4 does. One possible explanation of this apparently conflicting result could be that an unstable interaction between M23-AQP4 and α-syntrophin occurs in AQP4ex mouse. This is supported by the study of Neely et al. [24], demonstrating that a chemical cross-linking is necessary to detect the interaction between M23-AQP4 and α-syntrophin efficiently, indicating that the association between the two proteins, through the C-terminal sequence Ser-Ser-Val (SSV) of AQP4 and the PDZ domain of α-syntrophin is labile. Thus, in the brain of AQP4ex-KO mice, as a consequence of the AQP4-OAP mislocalization, this labile interaction also perturbs the committed perivascular localization of α-syntrophin, which may result in its instable interaction at the membrane facing the neuropile and, as a consequence its degradation will accellerate (Fig. 8). This condition does not occur in the global AQP4 null mouse, most likely because in the absence of OAPs this competitive and ineffective interaction does not occur, so that α-syntrophin could maintain its interaction with other proteins including the potassium channel KIR4.1 [5] and dystrobrevin [4] in astrocyte membranes facing blood vessels.

AQP4ex is also expressed at the basolateral membrane of the ependymal epithelium lining the ventricular cavities. Differently to the perivascular location, the ependymal expression of AQP4ex is not dependent on α-syntrophin. Interestingly, AQP4ex null mice have only slightly reduced AQP4 expression and grossly not mislocalized. This suggests that AQP4ex may have a distinct anchoring protein to target AQP4 to the particular membrane compartment of the ependymal epithelium. Another possibility is that the ependymal expression of AQP4ex may not require a specific anchoring protein since the tight junction hampers the diffusion to the apical side of the cell membrane.

### Role of AQP4ex in OAPs formation, stability and localization

A long lasting and still unresolved question concerns the role of the supramolecular organzation of AQP4 in OAPs. With the use of AQP4ex-KO animals we provide conclusive evidence that AQPex is included in AQP4-OAPs. More importantly, AQP4ex is required to address OAPs at the perivascular endfeet, as demonstrated by IF experiments, and that its absence rearranges OAPs size. Indeed, BN-PAGE experiments demonstrate that the OAP size of each identified pool was reduced in the AQPex-KO mouse without any substantial effect on the number of OAP pools. Moreover, CO-IP experiments demonstrate that AQP4ex interacts with the canonical isoforms. Altogether, these data suggest that AQPex-OAPs are likely not to exist as sole entities, which is compatible with the low expression level (~ 10%) of AQP4ex. Importantly, these slightly smaller OAPs in AQP4-ex null mouse were no longer localized at the perivascular site but they were evenly distributed in glial processes as noted by the increases in IF signal on the brain parenchyma. This redistribution confirms that AQP4ex is required for AQP4 polarization but also reveals that OAPs only consisting of M1/M23 are not capable of being retained at the perivascular pole. In addition, it can be concluded that brain parenchyma redistribution of AQP4 depends on the amount of AQP4ex expressed. Another important issue regarding OAPs formation concerns the role of the M1- and M23-AQP4 isoforms. Previous studies using transfected cells have suggested that OAPs made by M23 are largely immobile and this would be the main determinant factor for their localization at the perivascular pole [34]. Furthermore, the conclusion that the expression and organization of M1– and M23–AQP4 isoforms may provide a mechanism sufficient for AQP4 to be locally enriched in specific subcellular regions [35] is not supported by our *in vivo* data and needs to be to be reconsidered. Indeed, our results demonstrate that large M23/M1 -OAPs are not localized at the perivascular endfoot in AQP4ex mice indicating that it is not simply the size of OAPs or the M1/M23 ratio that determines their immobility and polarity at the perivascular pole, but rather the presence of AQP4ex in OAPs is the “*conditio sine qua non*” for the specific AQP4 location. In conclusion, although diffusional mobility of OAPs is highly dependent on the size [34], this effect is not at all or only minimally involved in AQP4 polarization at the vascular astrocyte endfeet.

### AQP4ex and NMO

As previously reported, NMO-IgG preferentially binds to AQP4-OAPs in transfected cells and to perivascular AQP4 in brain tissue [38]. However, it has also been reported that NMO serum [16] and monoclonal AQP4-specific recombinant antibody derived from an NMO patient (rAb-53) also bind to M1-AQP4 expressing cells [6], suggesting that the suprastructure organization may not be the only essential element for antibody binding. Our results show that the absence of AQP4ex abolishes the NMO-IgG staining in the perivascular region without a compensative staining of the processes facing the neuropile. Thus, although AQP4 is still largely present in forms of OAPs in AQP4ex-null mice, perivascular confinement is required for NMO-IgG to bind AQP4 *in vivo*. This implies that the conformational epitopes are generated by the interaction of additional components (i.e. syntrophin) requested to anchor the OAPs at the perivascular pole and affecting the AQP4 extracellular loop conformations. Thus, as a consequence, unlocking OAPs from the perivascular location would change the AQP4 extracellular loop conformation resulting in the modification of the epitope region. In conclusion, the specific protein interaction at the perivascular pole stabilizes the OAPs configuration and generates NMO-IgG epitopes.

These results lead to another important question: why in transfected cells are only the OAPs made by the M23 isoform recognized by NMO-IgG, while those expressed by the AQP4ex null mice are not? A possible explanation to this evident discrepancy is that the *in vivo* microenvironment may play a role in the quaternary structure of OAPs in such a way that subtly changing the OAP conformation would profoundly affect the NMO-IgG binding. Something similar occurs in skeletal muscle fast-twitch fibers where there are AQP4-OAPs that are much less recognized by NMO-IgG [33] compared to brain OAPs.

Therefore, we can state that *in vivo* the OAP is not the target per se of NMO-IgG but it is the specific OAP configuration at the perivascular astrocyte endfoot process.

Our data indicate AQP4ex to be crucial in the binding of NMO-IgG to its conformational epitope and strongly suggest that this isoforms may have a role in the pathogenesis of NMO. These also poses a limit on the use of transfected cells for mechanistic studies, and envisage the use of the AQP4 null mouse as a new animal model for studies in the NMO field and its use for testing specific pharmacologic treatments.

## Conclusion

In conclusion, this study provides the first direct evidence *in vivo* on the specific role of AQP4ex in AQP4 perivascular OAPs assembly and confinement, as well as its involvement as a structural component of the glial endfoot membrane protein functional unit. These findings suggest that the alteration of AQP4ex expression levels in astrocytes dysregulating AQP4 migration along their processes, becoming more enriched in astrocyte processes that normally have very low amounts of AQP4, may have a deleterious impact on brain water and ion homeostasis and are likely to result in neuronal function impairment. The absence in AQP4ex-KO mouse of an evident phenotype is not surprising considering that the same is occurring in other animal models in which AQP4 is similarly mislocalized (i.e. α-syntrophin and dystrophin null mice). Probably, a second event (inflammation, trauma, etc) may be necessary to determine an appearance of a more evident phenotype. In this regard, it is likely that AQP4ex may be involved in brain tumors where mislocalization of AQP4 has been found, such as in glioma.
